# Editorial: Advancements in trajectory optimization and model predictive control for legged systems

**DOI:** 10.3389/frobt.2022.1002552

**Published:** 2022-09-21

**Authors:** Enrico Mingo Hoffman, Chengxu Zhou, Matteo Parigi Polverini

**Affiliations:** ^1^ PAL Robotics, Barcelona, Spain; ^2^ University of Leeds, Leeds, United Kingdom; ^3^ Agility Robotics, Albany, CA, United States

**Keywords:** trajectory optimization, model predictive control, legged robots, humanoid robots, whole-body control

In recent years we witnessed the proliferation of legged systems as advanced robotic platforms, which could be potentially employed in the near future on a huge spectrum of useful tasks. This is not only related to the success of quadrupeds, e.g. for inspection tasks (Bouman et al. (2020); Gehring et al. (2021)), but also to the increased capabilities of humanoid bipedal systems enabling operation in real-world settings (Agility Robotics, 2021; BostonDynamics (2021a)). To achieve such a level of autonomy, the use of advanced control and planning tools has been fundamental, in particular those allowing for the full exploitation of the dynamics of the system within a prediction horizon. In this respect, Trajectory Optimization (TO) and Model Predictive Control (MPC), among other methods, have proved to be highly effective on legged robotics platforms for a multitude of different tasks, including locomotion and manipulation Koenemann et al. (2015); Neunert et al. (2018); Di Carlo et al. (2018); Kuindersma et al. (2016); Belli et al. (2021). While TO is commonly used to plan complex open-loop trajectories over a long prediction horizon, MPC enables fast re-planning and feedback stabilization, over a shorter horizon. Thanks to the combination of these techniques, legged systems have been able to successfully demonstrate complex high-level skills like loco-manipulation in complex environments, agile locomotion, parkour, and much more in the years to come. However, it still remains an open question how to effectively employ TO and MPC on real legged systems. This requires smart formulations of contact-constrained robot dynamics, convenient models of the environment, as well as computationally efficient and real-time optimization algorithms.

The aim of this Research Topic has been to inform the robotics research community on the newest findings and future directions in TO and MPC applied to legged systems. In particular, we collected different contributions concerning recent applications of TO and MPC on legged systems related to climbing, dynamic locomotion and running, whole-body manipulation, robust formulation of contacts under modeling uncertainties, and software frameworks.

The first paper in our Research Topic, by L. Drnach et al. proposes a novel framework to account for contact uncertainty in TO. The authors developed *chance complementarity constraints* to convert the stochastic constraints into deterministic constraints showing that the chance constraints can mediate a trade-off between feasibility and robustness.

Locomotion in simulated bipedal systems is addressed in two works by K. Wan et al. and F. M. Smaldone et al. respectively. In particular, the first work proposes a fast MPC based on a Quadratic Programming (QP) formulation for automatic footstep placement; the computed controls are consecutively tracked by whole-body inverse dynamics. The locomotion scheme is validated in simulation using a novel platform, called SLIDER, walking blindly in various uneven terrain. The second work presents an MPC for running and walking based on the Variable-Height Inverted Pendulum (VH-IP) model (Koolen et al. (2016)) and adaptive footstep placement. The nonlinearity of the VH-IP is handled by splitting the gait generation into two consecutive stages, both requiring a QP to be solved. The proposed method is validated via dynamic simulations on the full-scale humanoid robot HRP-4 (Kaneko et al. (2011)), and it is experimentally validated on the small-sized robot OP3 (ROBOTIS (2022)).

In the two works from F. Aller et al. and P. Q. Lee et al. TO is applied to the REEM-C robot (PAL Robotics (2020)) to generate trajectories for a whole-body loco-manipulation task and dexterous manipulation, respectively. In the first work, sit-to-stand trajectories are optimized using optimal control, generating dynamic and human-like motions. Based on the REEM-C sensor data, a unified bench-marking procedure is presented based on two different experimental protocols. In the second paper, the buzzwire task is used to benchmark manipulation capabilities.

The work by F. Ruscelli et al. introduces a novel software framework named *Horizon* for TO tailored to robotics applications. Horizon is based on CasADi (Andersson et al. (2019)) and permits easily setting up and solving complex TO problems using different approaches and solvers. The authors present contact rich dynamic motions on different types of platforms, including KANGAROO (Roig et al. (2022)) and Spot^®^ (BostonDynamics (2021b)) together with a real demonstration on the CENTAURO robot (Klamt et al. (2019)).

Finally, the paper from J. Lee et al. proposes a robust planning and control framework for climbing robots that provides robustness resistance to slippage in unknown environments. The framework combines TO for the center of mass position with re-planning, according to the uncertainties related to the environment; a whole-body controller is able to adjust the ground reaction force distribution in real-time. The proposed method is tested in simulation using a novel magnetic-legged climbing robot, Magneto.

We conclude by acknowledging the important contribution made by the reviewers of the articles submitted to this collection. They played a significant role in the high quality of the articles presented here. We hope that researchers and practitioners will enjoy this Research Topic and will be fascinated and inspired, as we are, by reading these articles.
